# Prostate-Specific Membrane Antigen Expression in Patients with Primary Prostate Cancer: Diagnostic and Prognostic Value in Positron Emission Tomography-Prostate-Specific Membrane Antigen

**DOI:** 10.3390/curroncol31080311

**Published:** 2024-07-24

**Authors:** Omar Tayara, Sławomir Poletajew, Wojciech Malewski, Jolanta Kunikowska, Kacper Pełka, Piotr Kryst, Łukasz Nyk

**Affiliations:** 1 Second Department of Urology, Centre of Postgraduate Medical Education, 02-511 Warsaw, Poland; slawomir.poletajew@cmkp.edu.pl (S.P.); wojtek.malewski@gmail.com (W.M.); piotr.kryst@onet.pl (P.K.); ukinyk@poczta.fm (Ł.N.); 2 Department of Nuclear Medicine, Medical University of Warsaw, 02-091 Warsaw, Poland; jolanta.kunikowska@wum.edu.pl (J.K.); kacper.pelka@wum.edu.pl (K.P.); 3 Department of Methodology Laboratory, Centre for Preclinical Research, Medical University of Warsaw, 02-091 Warsaw, Poland

**Keywords:** prostate-specific antigen (PSA), prostate-specific membrane antigen (PSMA), positron emission tomography (PET), computed tomography (CT), magnetic resonance imaging (MRI), Food and Drug Administration (FDA), Carbon-11 Choline (C-11 Choline), 18F-fluoro-pyridine-3-carbonyl)-amino]-pentyl}-ureido)-pentanedioic acid (DCFPy), seminal vesicle invasion (SVI), Gleason score (GS), radiomic features (RF)

## Abstract

Prostate cancer represents a significant public health challenge, with its management requiring precise diagnostic and prognostic tools. Prostate-specific membrane antigen (PSMA), a cell surface enzyme overexpressed in prostate cancer cells, has emerged as a pivotal biomarker. PSMA’s ability to increase the sensitivity of PET imaging has revolutionized its application in the clinical management of prostate cancer. The advancements in PET-PSMA imaging technologies and methodologies, including the development of PSMA-targeted radiotracers and optimized imaging protocols, led to diagnostic accuracy and clinical utility across different stages of prostate cancer. This highlights its superiority in staging and its comparative effectiveness against conventional imaging modalities. This paper analyzes the impact of PET-PSMA on prostate cancer management, discussing the existing challenges and suggesting future research directions. The integration of recent studies and reviews underscores the evolving understanding of PET-PSMA imaging, marking its significant but still expanding role in clinical practice. This comprehensive review serves as a crucial resource for clinicians and researchers involved in the multifaceted domains of prostate cancer diagnosis, treatment, and management.

## 1. Introduction

Prostate cancer remains one of the most prevalent malignancies affecting men globally, with varying prognoses based on stage and molecular characteristics at diagnosis [1]. Driven by significant advancements in diagnostic and therapeutic technologies, the landscape of prostate cancer management is rapidly evolving. Diagnosis typically involves a digital rectal examination and prostate-specific antigen (PSA) testing, followed by a biopsy confirmation. However, traditional imaging techniques like magnetic resonance imaging (MRI) face challenges in diagnosing prostate cancer due to limited sensitivity and specificity. Hence, there is a growing need for alternative diagnostic methods.

Among the biomarkers for prostate cancer, prostate-specific membrane antigen (PSMA) has emerged as a pivotal target for imaging and therapeutic approaches [2]. PSMA, a transmembrane protein, is significantly overexpressed in primary prostate cancer tissues, as well metastases, especially in lymph nodes and bones [3]. Hence, PSMA is deemed a viable target for positron emission tomography (PET) imaging in prostate cancer.

Imaging plays a crucial role in facilitating early diagnosis and treatment, assessing eligibility for therapy, monitoring treatment response, and conducting surveillance for prostate cancer. Anatomical imaging techniques such as transrectal ultrasound (TRUS), computed tomography (CT), and MRI are used alongside functional imaging modalities like position emission tomography (PET) [4]. PET imaging using PSMA ligands has revolutionized the management of prostate cancer, offering superior sensitivity and specificity in detecting metastatic disease burden [5]. The introduction of PSMA-PET imaging has led to significant improvements in the staging, restaging, and treatment planning for prostate cancer patients, and PSMA-based theranostics have recently gained approval from the Food and Drug Administration (FDA) [6].

Its ability to accurately detect disease recurrence at low PSA levels and guide the selection of patients for targeted therapies underscores its diagnostic and prognostic value. Moreover, the integration of PSMA-PET imaging into clinical guidelines as a first-line imaging option for primary staging of prostate cancer reflects its growing importance in the personalized management of prostate cancer [7]. Nonetheless, despite these advancements, the heterogeneous nature of PSMA expression among prostate cancer patients poses challenges, necessitating a comprehensive review to synthesize existing evidence and guide future research endeavors.

This review will examine PSMA expression in prostate cancer through PET-PSMA imaging, emphasizing the need for a nuanced understanding of its clinical implications and guiding future research in optimizing PSMA-targeted diagnostics and therapeutics.

## 2. The Biological Underpinnings of PSMA as a Target in Prostate Cancer

PSMA, an extensively studied protein in the context of prostate cancer, holds multifaceted significance due to its diverse structural and functional attributes. Also recognized as glutamate carboxypeptidase II or folate hydrolase 1, PSMA is a type II transmembrane glycoprotein with folate hydrolase activity [8]. This enzymatic function intricately regulates various biological processes critical for tumor growth and survival, including nutrient uptake and the modulation of intracellular signaling pathways. Notably, in prostate cancer’s intricate molecular scene, PSMA’s enzymatic activity extends to folate metabolism and polyglutamate substrate cleavage, delineating its pivotal role in cancer biology. Specifically, PSMA emerges as a key facilitator; it plays a crucial role in glutamate cleavage along the PI3K/AKT growth pathway in prostate cancer [9].

Moreover, the differential expression pattern of PSMA, characterized by its overexpression in prostate cancer cells compared to normal prostate tissue, serves as a foundational basis for its utility in both diagnostic and therapeutic interventions. This heightened expression is not only indicative of cancer aggressiveness but also underscores the potential of PSMA as a robust target for precision medicine approaches in prostate cancer management. Beyond its canonical association with prostate tissue, PSMA expression extends to various extra-prostatic tissues, encompassing the salivary and lacrimal glands, proximal tubules of the kidney, liver, small bowel, colon, and nonmyelinated ganglia [10]. This widespread expression profile, coupled with its presence in certain non-prostatic benign and malignant conditions, accentuates the complexity of PSMA’s physiological and pathological roles [11].

In the context of prostate cancer progression and metastasis, PSMA emerges as a multifaceted player, with its expression levels correlating with tumor grade, stage, and prognosis. Its involvement in facilitating tumor cell invasion through diverse mechanisms, including its enzymatic activity and intracellular signaling pathway modulation, further underscores its significance in prostate cancer biology [12]. Additionally, emerging evidence implicates PSMA in angiogenesis, thereby broadening its scope of influence in tumor microenvironment modulation [13]. However, the differential landscape of PSMA expression warrants cautious interpretation; some prostate cancer cells express low PSMA expression, as they may lose this expression, along with other receptors [14]. Particularly, in variant histopathologies such as small-cell neuroendocrine and ductal prostate cancer, PSMA expression may be lower or absent [15]. Consequently, comprehensive assessment strategies, including cross-referencing with imaging modalities like PET scans alongside traditional histopathological analyses, become imperative for accurate diagnosis and treatment planning.

**Figure 1:**
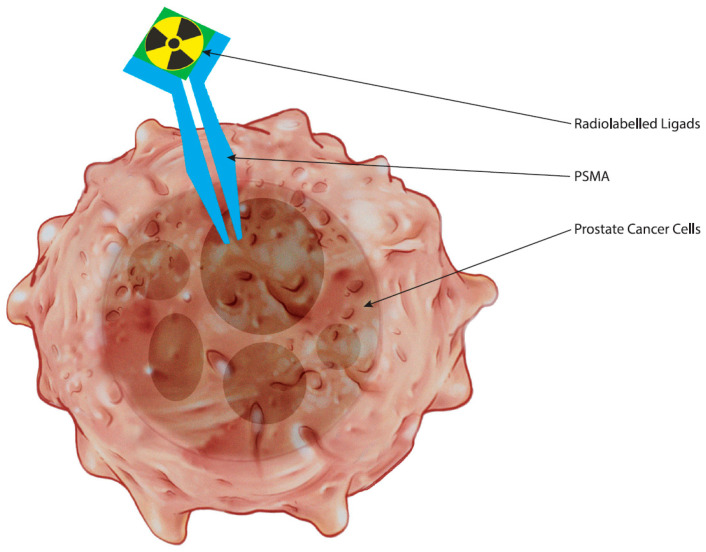
The figure illustrates prostate cancer cell with a PSMA receptor and a radiolabeled ligand binding to it.

As a cell surface enzyme, PSMA facilitates rapid internalization of bound ligands, which is exploited by radiolabeled small molecular ligands used in PET imaging (Figure 1). These ligands, which clear from the blood more rapidly than larger molecules, enhance the target-to-background ratio, providing clearer and more accurate imaging results. This biological feature of PSMA is pivotal, as FDA-approved radiotracers leverage this mechanism to improve the detection and characterization of prostate cancer. Such precise targeting reflects the broader biological roles of PSMA in prostate cancer, including its involvement in tumor cell invasion and angiogenesis, thereby confirming its significance in imaging for diagnostic, prognostic, and therapeutic markers, thus opening avenues for tailored therapeutic interventions and personalized management strategies aimed at improving patient outcomes in prostate cancer.

## 3. Advances in PET-PSMA Imaging Technologies and Methodologies

**Figure 2:**
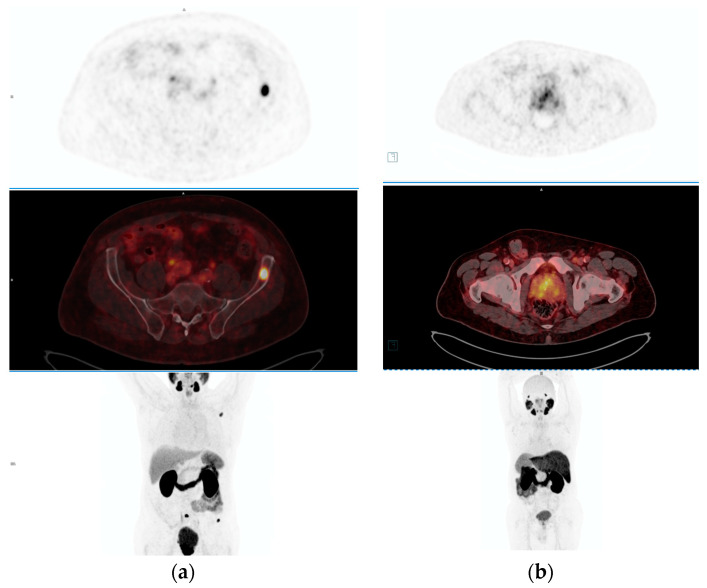
The figure contrasts PSMA-PET scans between PSMA-positive and PSMA-negative cases in prostate cancer diagnosis. PSMA-positive scans (**a**) reveal intense radiotracer uptake, indicating cancerous cell presence, while PSMA-negative scans (**b**) show minimal uptake, suggesting either the absence of cancer or lower PSMA expression.

PET imaging technology has significantly transformed the landscape of prostate cancer diagnosis and management, particularly in the realm of staging and detecting suspected metastatic disease. Unlike conventional imaging modalities such as CT and MRI, which primarily rely on lymph node size and morphology and often exhibit suboptimal sensitivity, PET imaging operates on a fundamentally different principle [16]. It involves the administration of radiotracers that emit positrons, which interact with electrons within the body, resulting in the generation of two photons moving in opposite directions. PET scanners then detect these photons, enabling the reconstruction of three-dimensional images that highlight areas of increased tracer uptake, thus providing invaluable insights into abnormal tissue metabolism (Figure 2). The advent of PET imaging has revolutionized prostate cancer diagnosis and management by offering enhanced sensitivity and specificity.

The evolution of PET tracers for prostate cancer imaging has further augmented the capabilities of PET technology in this domain. Fluorine-18 Fluciclovine (Axumin) was one of the first PET tracers approved for prostate cancer imaging, significantly enhancing the diagnostic accuracy of conventional imaging modalities [17]. The emergence of PSMA-targeted imaging agents exploits this elevated expression to delineate the dissemination of prostate cancer with exceptional sensitivity and specificity. Leveraging the substantial upregulation of PSMA in prostate cancer cells, these agents facilitate superior detection of prostate cancer compared to alternative imaging tracers. A radiolabeled PSMA ligand is considered a more suitable tracer for detecting distant metastatic disease in prostate cancer compared to other metabolic prostate cancer PET alternatives like fluciclovine [18]. The emergence of 68Ga-PSMA-11 as an FDA-approved PET tracer represents a significant milestone in prostate cancer imaging, enabling the highly sensitive and specific detection of prostate cancer lesions [17]. More recently, the approval of 18F-fluoro-pyridine-3-carbonyl)-amino]-pentyl}-ureido)-pentanedioic acid (DCFPy) has introduced a new dimension to PET imaging, offering potential advantages over existing tracers such as 68Ga-PSMA-11. With a longer half-life and lower positron energy level, DCFPy exhibits improved count statistics and spatial resolution, further improving the precision of prostate cancer diagnosis and staging [19]. Studies using both 68Ga-PSMA-11 and 18F-DCFPyL PET/CT have reported similar results, with higher sensitivity achieved upon excluding small nodal deposits below PET detection limits [20,21]

PET-PSMA imaging outperforms conventional imaging modalities in detecting nodal and distant metastatic disease in patients with intermediate and high-risk prostate cancer [22]. A prospective randomized phase III PSMA trial provides level 1 evidence supporting the use of 18F-DCFPyL or 68Ga-PSMA-11 PSMA-PET for staging prior to definitive primary therapy [23]. 68Ga-PSMA-11 PSMA-PET/CT exhibits higher sensitivity, specificity, and accuracy compared to conventional imaging, leading to lower false-positive rates and improved staging accuracy [24].

As various PSMA-targeted radiotracers have been developed and validated for clinical use in prostate imaging, the selection of PSMA ligands and PET radionuclides can significantly affect diagnostic outcomes and biodistribution. This highlights the importance of thorough documentation and standardized reporting in imaging studies to ensure consistent results. Accurate interpretation of PET imaging findings in prostate cancer requires a comprehensive understanding of various factors, including the choice of radiotracer, elapsed time since injection, and standardized reporting criteria. Different PSMA peptides, such as 68Ga-PSMA-11 and 18F-PSMA-1007, have demonstrated equivalent clinical value but may exhibit differences in biodistribution and diagnostic thresholds. For instance, a study revealed that 18F-PSMA-1007 demonstrated higher uptake within involved nodes and distant metastases compared to 68Ga-PSMA-11 but had more equivocal results for bone lesions [25]. Thresholds for PSMA intensity indicating metastatic disease may vary depending on the PSMA peptide utilized, with considerations like SUVmax cutoffs tailored accordingly [26].

Different techniques for performing the test or radioisotopes used yield different sensitivities and specificities for disease detection. An example is the search for metastatic lymph nodes. For classical imaging methods, the sensitivity was 0.42 (0.26–0.56 95% CI) and 0.39 (0.22–0.56 95% CI) and specificity was 0.82 (0.8–0.83 95% CI) and 0.82 (0.79–0.83 95% CI) for CT and MRI, respectively [16]. Indium-111 capromab pendetide (the earliest FDA-approved PSMA-ligand radiotracer) had 69% sensitivity and 58% specificity for diagnosing distant metastatic disease [17]. PET/CT imaging with PSMA showed the highest sensitivity in the evaluation of lymph nodes and bone lesions for biochemical recurrence, but sensitivity depended on PSA levels. Diagnostic accuracy to detect pelvic nodal or distant metastatic disease in high-risk patients for 68Ga-PSMA-11 showed a sensitivity 85% (74–96%) and specificity 98% (95–100%) [22]. 18F-PSMA-1007 showed similar values for nodal lesion detection as 68Ga-PSMA-11 [25]. The diagnostic performance of 18F-DCFPyL-PET/CT for the determination of pelvic lymph node metastases in high-risk patients had a sensitivity up to 62.9 (46.9, 78.9) and a specificity up to 98.9 (96.0, 100) [21].

Precise data analysis is essential for interpreting PET-PSMA imaging findings and guiding clinical decision-making. Clinicians and imaging specialists must understand current literature to estimate the likelihood of significant equivocal findings and provide conclusive interpretations. Reporting guidelines, such as the European Association of Nuclear Medicine (EANM) criteria, PROMISE criteria, and PSMA-RADS, ensure consistent interpretation and facilitate communication between clinicians, imaging specialists, and referring physicians [27,28,29]. Structured reports include detailed information on imaging methodology, findings categorized according to miTNM classification, and quantitative data such as lesion count and PSMA expression [30]. PSMA-PET/CT is increasingly recognized as a first-line imaging option for primary staging of prostate cancer, supported by organizations like the EANM, The Society of Nuclear Medicine and Molecular Imaging (SNMMI), and the National Comprehensive Cancer Network (NCCN) [7,31]. The European Association of Urology (EAU) recommends incorporating PSMA-PET/CT for its enhanced accuracy in staging intermediate- and high-risk prostate cancer [32]. However, they promote cautious interpretation, and further research is necessary to fully integrate this modality into treatment protocols and to understand its impact on long-term patient outcomes.

## 4. Diagnostic Accuracy and Clinical Utility of PET-PSMA across Different Stages of Prostate Cancer

**Figure 3:**
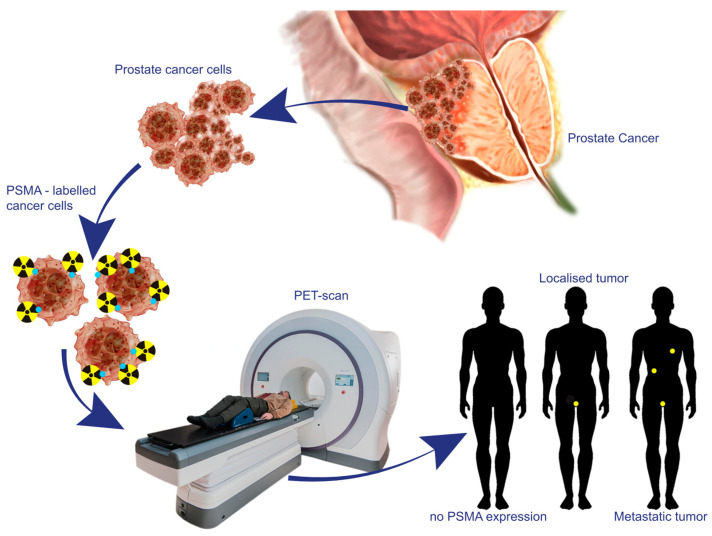
The schematic diagram demonstrates the mechanism behind PSMA PET imaging for diagnosing prostate cancer. By utilizing PSMA receptor binding proteins on cancer cell surfaces, labeled radioisotopes are internalized, enabling precise visualization via PET. This technique allows the detection of localized or metastatic cancer with high accuracy.

PET-PSMA imaging technology has emerged as an indispensable tool in the initial diagnosis of prostate cancer, playing a pivotal role in influencing treatment decisions through accurate staging (Figure 3). It demonstrated superior diagnostic accuracy compared to conventional imaging modalities such as CT, MRI, and bone scintigraphy in detecting nodal and distant metastatic disease [22]. Kalpara et al. highlighted a complementary accuracy of PSMA PET/CT and mpMRI in detecting prostate cancer lesions [33], while some studies have suggested PSMA PET/CT outperforms mpMRI in certain patient groups [34]. Additionally, PSMA PET/CT-targeted biopsy offers a higher detection rate compared to systemic biopsy and mpMRI-targeted biopsy, although further studies are warranted to establish clear patient selection criteria and determine its feasibility and safety as a biopsy-free diagnostic tool for prostate cancer. It is crucial to acknowledge that while PSMA can accumulate in inflammatory conditions, potentially leading to elevated levels and false positives, the combination of PSMA PET/CT with mpMRI significantly improves the detection of prostate cancer lesions. This integrated approach not only reduces the likelihood of missing tumors but also minimizes the number of unnecessary biopsies [35].

Furthermore, PET-PSMA imaging significantly aids in the staging of prostate cancer, facilitating precise planning of curative-intent surgeries or radiotherapies. PET-PSMA/CT has received level 1 evidence backing its use for staging before definitive primary therapy [22]. Research, such as a meta-analysis conducted by Jeet et al. has demonstrated that PSMA PET/CT induces a change in clinical management for approximately 28% of patients with primary prostate cancer compared to conventional imaging during primary staging [36]. PET-PSMA imaging enhances the effectiveness of preoperative selection for radical prostatectomy by precisely delineating the extent of the primary tumor and detecting regional and distant metastases in patients with elevated PSA levels suspected of prostate cancer. A recent study found that using PSMA PET/CT for primary staging led to a significant reduction in the risk of biochemical recurrence in patients selected for radical prostatectomy compared to conventional imaging [37]. Moreover, in high-risk prostate cancer cases, PET-PSMA imaging can prompt modifications in treatment plans, transitioning from localized interventions to systemic therapies upon the early identification of metastatic disease [38].

Studies have indicated that 68Ga-PSMA-11 PET/CT is size-criteria independent and detects very small lesions with superior diagnostic accuracy [39]. However, the study by Jeet et al. showed that PSMA PET exhibited limitations in detecting small deposits. However, they reported a lower sensitivity, likely due to the inclusion of retrospective clinical studies that often have pre-surgery selection bias [36]. This therapeutic approach highlights the translational potential of understanding PSMA’s biological role, from a diagnostic biomarker to a therapeutic target, promising a paradigm shift in prostate cancer treatment.

Prostate cancer staging encompasses the evaluation of the primary tumor (T), lymph node involvement (N), and distant metastasis (M), which collectively determine cancer severity and guide treatment decisions. While both PSMA PET/CT and mpMRI demonstrate effectiveness in T-staging, studies have indicated that mpMRI exhibits higher overall detection rates for extra-prostatic extension (EPE) and seminal vesicle invasion (SVI) compared to PSMA PET/CT [37]. Moreover, mpMRI shows superior sensitivity, specificity, positive predictive value, and negative predictive value for SVI, albeit not for EPE, when compared to PSMA PET/CT [38]. On the other hand, PSMA PET/MRI demonstrates enhanced accuracy in staging prostate cancer, particularly for predicting T2, T3a, and T3b stages [39]. Additionally, in primary N staging, PSMA PET/CT surpasses traditional imaging methods, which often prove inadequate in newly diagnosed prostate cancer cases [40]. Lymph node metastases are frequently observed in approximately one-third of high-risk prostate cancer patients primarily staged using PSMA PET/CT [41].

While limited evidence exists supporting the assessment of treatment response using PSMA-PET, its potential lies in accurately staging primary prostate cancer and guiding therapeutic decisions, particularly before radical prostatectomy [39]. Simultaneous 18F-PSMA-1007 PET/MRI demonstrated clinical feasibility and reproducibility in imaging prostate cancer patients, with PET/MRI achieving optimal co-registration results [42]. Additionally, 68Ga-PSMA-11 PET/MRI has been proposed as the ideal imaging modality for staging prostate cancer patients, with greater diagnostic accuracy for localization compared to multiparametric MRI or PET alone [28,43]. Despite the presumed superior ability of PET/MRI to assess the prostate and its surrounding area compared to PET/CT, compared to mpMRI, PET/MRI is only ahead of mpMRI in sensitivity for detecting local lesions and follicular involvement: 78.7 (69.3–85.8) vs. 52.9 (43.3–62.3) and 66.7 (48.4–88.0) vs. 51.0 (33.2–68.8), respectively, and it is inferior in specificity to mpMRI 82.2 (71.3–89.5) vs. 86.2 (76.2, 92.4) and 92.4 (86.8–95.7) vs. 96.6 (92.0–98.6) [43]. For the diagnosis of localized lesion or follicular infiltration, PET/CT is inferior in sensitivity and specificity to any mpMRI category. PSMA PET/MRI combines structural, multiparametric functional, and molecular information, enhancing diagnostic performance for TNM staging. However, PSMA PET has shown superiority over MRI in identifying distant metastases, with PSMA PET scans revealing previously unknown nodal involvement in a significant percentage of patients [44]. Despite the ongoing debate regarding the correlation between PSMA PET signal and Gleason score (GS), recent research has focused on investigating the discriminatory ability of PSMA PET-derived radiomic features (RF) in intraprostatic tumor characterization, prostate cancer discrimination, and GS and lymph node status prediction [45]. The study demonstrated the feasibility and efficacy of PSMA PET RF in improving prognostic accuracy and guiding personalized treatment strategies, ultimately enhancing clinical management and reducing unnecessary interventions. A study by Solari et al. [46] assessed the efficacy of handcrafted RF, manually derived quantitative imaging features from pre-therapeutic PSMA PET/MRI, in predicting postsurgical GS for primary prostate cancer. By integrating imaging features with clinical parameters, results revealed that combining features from PSMA PET and apparent diffusion coefficient maps surpassed single-modality and baseline models using clinical data alone. The hybrid radiomics model showed superior post-surgical GS prediction compared to biopsy GS [47]. A pioneering randomized phase 3 trial investigated whether the incorporation of PSMA PET/CT molecular imaging could enhance outcomes in prostate cancer patients undergoing definitive radiotherapy by refining patient selection and customizing radiotherapy plans [23].

In summary, PET PSMA imaging has significantly improved the diagnostic precision at various stages of prostate cancer, ranging from initial diagnosis to recurrence detection and qualifying for radioisotope treatment. Its integration into prostate cancer care protocols has facilitated the development of more personalized, targeted, and effective treatment strategies, thus playing a crucial role in enhancing patient outcomes in prostate cancer therapy.

## 5. The Predictive Value of PSMA Expression in Response to Therapy as Determined by PET-PSMA Imaging

The correlation between PSMA expression levels and treatment response in prostate cancer underscores the pivotal role of PET-PSMA imaging in stratifying patients based on their risk profile and guiding personalized treatment approaches. The use of PSMA-PET/CT for outcome prediction, especially regarding overall survival, is an area of active research in the field of prostate cancer management [48]. Elevated PSMA expression, often observed through PET-PSMA imaging, tends to correlate with more aggressive disease states and worse prognosis, highlighting the utility of PET-PSMA in aiding precise staging and therapeutic decisions [49]. However, the significant heterogeneity in PSMA expression within primary tumors and among patients complicates the understanding of this relationship, necessitating further research for a comprehensive assessment [46]. Seifert and colleagues selected patients with higher PSMA expression, as measured by SUVmax, for 177Lu-PSMA-617 therapy. These patients responded better to the treatment and demonstrated longer survival [50]. Larger tumor volume on PSMA-ligand PET scans was associated with poorer outcomes [51]. However, limitations include many studies’ relatively small sample sizes and the lack of standardized assessment methods for PSMA-ligand PET/CT scans.

The integration of PET-PSMA imaging into prostate cancer management significantly impacts treatment decision-making by facilitating personalized approaches tailored to disease severity and aggressiveness, ultimately leading to more precise and effective management of prostate cancer across different clinical stages. Changes in PSMA expression levels post therapy serve as early biomarkers of treatment efficacy, allowing clinicians to monitor disease response and modify treatment plans accordingly. Despite challenges in standardization and further research requirements, PET-PSMA imaging represents a significant advancement in prostate cancer management, offering superior diagnostic capabilities and invaluable insights into treatment response dynamics, ultimately leading to improved patient outcomes and quality of life. Moreover, the diverse clinical presentations of prostate cancer underscore the importance of leveraging advanced imaging modalities like PET-PSMA alongside traditional techniques to comprehensively evaluate disease characteristics and tailor treatment strategies accordingly. Prostate cancer presents a diverse array of clinical scenarios, from early localized disease amenable to curative treatments such as surgery or radiotherapy to advanced metastatic disease requiring systemic therapies like hormones, radiation, or chemotherapy [52]. Central to the management of prostate cancer is the targeting of the androgen receptor signaling pathway, which plays a pivotal role in driving prostate cancer cell proliferation and growth. PSMA expression is a potential biomarker for androgen receptor signaling, offering a noninvasive method for its assessment [53]. Traditional imaging modalities, such as MRI for assessing the effects of local therapies and CT and bone scans for systemic treatments, have been foundational in evaluating treatment responses [54]. However, the emergence of newer imaging techniques, particularly PET-PSMA, has revolutionized this landscape by offering enhanced capabilities in assessing therapeutic responses across different clinical stages of prostate cancer. Hofman et al. revealed that PSMA PET-CT led to alterations in surgical techniques based on imaging findings [22]. Additionally, Karagiannis et al. demonstrated significant modifications in radiotherapy treatment plans for the bigger half of patients following [38]. Collectively, these studies highlight how PSMA PET-CT imaging informs personalized treatment strategies, ensuring more precise and effective management of prostate cancer. Changes in PSMA expression levels post therapy serve as early biomarkers of treatment efficacy, allowing clinicians to monitor disease response and modify treatment plans accordingly. A study indicated that low PSMA expression may negatively impact patient outcomes with Lu-PSMA therapy, highlighting the need for further research to determine the optimal level of PSMA expression for effective treatment [50].

The widespread adoption of PET-PSMA imaging in clinical practice necessitates standardized criteria for interpretation and further research into its behavior across different treatment modalities. Despite these challenges, PET-PSMA imaging represents a significant advancement in prostate cancer management, offering superior diagnostic capabilities and invaluable insights into treatment response dynamics, ultimately leading to improved patient outcomes and quality of life.

## 6. Limitations and Future Directions

The utilization of PET-PSMA imaging in patients with primary prostate cancer has yielded significant insights into its diagnostic and prognostic value, as well as its implications for clinical practice. Several key findings have emerged through a comprehensive review of available literature, integrating insights from recent studies, meta-analyses, and reviews. PET-PSMA demonstrates superior sensitivity and specificity in detecting primary prostate cancer lesions compared to conventional imaging modalities. Mapelli et al. conducted a systematic review and meta-analysis assessing the diagnostic accuracy of PSMA PET/MRI in primary prostate cancer assessment, demonstrating superior sensitivity and specificity compared to multiparametric MRI alone [55]. PET-PSMA imaging offers valuable information regarding disease extent, lymph node involvement, and distant metastases, aiding in accurate staging and treatment planning [22]. A meta-analysis focused on the interobserver variability in 68Ga-PSMA-11 PET/CT-MR imaging, revealing substantial agreement among readers in interpreting tumoral lesions in different anatomical locations [56]. A meta-analysis focused on the diagnostic accuracy of PSMA PET in tumor staging of newly diagnosed prostate cancer, demonstrating high accuracy for intraprostatic tumors and extra-prostatic extension, particularly with PSMA PET/MR [57].

Furthermore, PET-PSMA has prognostic implications, with studies showing correlations between PSMA expression levels and disease outcome, as well as treatment response [48]. This highlights its potential as a biomarker for risk stratification and personalized management strategies. Another systematic review and meta-analysis evaluated the impact of PSMA PET on patient management and outcomes in cases of biochemical recurrence after primary therapy, highlighting a substantial proportion of patients with management changes and favorable biochemical response rates following PSMA PET-directed therapy [36]. The integration of PET-PSMA imaging into clinical practice carries significant implications for the management of primary prostate cancer. Its ability to provide detailed anatomical and functional information can guide treatment decision-making, leading to more tailored and effective therapeutic approaches. PET-PSMA facilitates the identification of occult metastases, enabling early intervention and potentially improving patient outcomes [58]. Additionally, it offers a non-invasive means of monitoring treatment response, allowing for timely adjustments to therapy. These recent studies collectively underscore the growing importance of PSMA-based PET imaging in prostate cancer management, offering improved diagnostic accuracy, impact on patient management, and potential for guiding treatment decisions. Incorporating these findings into clinical practice can enhance the precision of prostate cancer staging, facilitate personalized treatment strategies, and improve patient outcomes. However, further research and prospective studies are warranted to validate these findings and address existing gaps in knowledge, ensuring the optimal integration of PSMA PET imaging into routine clinical care for prostate cancer patients.

Despite its promise, PET-PSMA imaging is not without limitations and challenges. False positives and negatives can occur due to physiological uptake and variations in PSMA expression levels, necessitating careful interpretation of findings [59,60]. Accessibility of PET-PSMA technology may be limited in certain regions, hindering its widespread adoption in clinical practice. Moreover, challenges related to image interpretation and standardization across centers pose obstacles to achieving consistency and reliability in results [61,62]. To address the limitations and challenges associated with PET-PSMA imaging, future research efforts should focus on several key areas. This includes the development of standardized imaging protocols and interpretation criteria to ensure uniformity across centers. Furthermore, investigations into the clinical utility of PET-PSMA as a prognostic biomarker and its potential role in guiding targeted therapies are warranted. Prospective studies are needed to validate its predictive value in different clinical scenarios and assess its impact on patient outcomes.

In conclusion, the utilization of PET-PSMA imaging represents a pivotal advancement in the realm of prostate cancer diagnosis and management. Through its superior sensitivity and specificity, PET-PSMA imaging offers invaluable insights into disease staging, treatment planning, and prognostic assessment. By guiding personalized treatment approaches tailored to individual patient profiles, PET-PSMA imaging has significantly improved the precision and efficacy of prostate cancer care. Despite encountering challenges such as false positives and limited accessibility, recent studies have underscored its diagnostic accuracy and clinical relevance. Looking ahead, further research endeavors are warranted to address these challenges, refine imaging protocols, and explore novel applications, thus solidifying PET-PSMA imaging as a cornerstone in the routine clinical management of prostate cancer. The continued integration and optimization of PET-PSMA imaging hold great promise for enhancing patient outcomes and quality of life in the ever-evolving landscape of prostate cancer therapy.
